# Procyanidin B2 Attenuates Nicotine-Induced Hepatocyte Pyroptosis through a PPARγ-Dependent Mechanism

**DOI:** 10.3390/nu14091756

**Published:** 2022-04-22

**Authors:** Jia Liu, Qinyu Yao, Xinya Xie, Qi Cui, Tingting Jiang, Ziwei Zhao, Xiong Du, Baochang Lai, Lei Xiao, Nanping Wang

**Affiliations:** 1 Cardiovascular Research Center, School of Basic Medical Sciences, Xi’an Jiaotong University, Xi’an 710061, China; jeven_liu@xjtu.edu.cn (J.L.); qinyu.yao@xjtu.edu.cn (Q.Y.); xiexinya916@stu.xjtu.edu.cn (X.X.); duxiong0414@stu.xjtu.edu.cn (X.D.); laibc@xjtu.edu.cn (B.L.); xiaolei0122@xjtu.edu.cn (L.X.); 2 Key Laboratory of Environment and Genes Related to Diseases, Xi’an Jiaotong University, Ministry of Education of China, Xi’an 710061, China; 3 Advanced Institute for Medical Sciences, Dalian Medical University, Dalian 116044, China; qicui1019@163.com (Q.C.); jiangtdl@163.com (T.J.); curry15zw@163.com (Z.Z.); 4 Health Science Center, East China Normal University, Shanghai 200241, China

**Keywords:** Procyanidin B2, nicotine, pyroptosis, peroxisome proliferator-activated receptor-γ, hepatocyte

## Abstract

Procyanidin B2 (PCB2), a natural flavonoid, has been demonstrated to exert anti-oxidation and anti-inflammatory effects on hepatic diseases. Increasing evidence shows the hepatoxicity of nicotine. However, whether PCB2 protects against nicotine-induced hepatoxicity and the underlying mechanisms remains uncharacterized. Here, we reported that nicotine promoted hepatocyte pyroptosis, as evidenced by the elevation of propidium iodide (PI)-positive cells, the activation of Caspase-1 and gasdermin D (GSDMD), the enhanced expression of NOD-like receptor containing pyrin domain 3 (NLRP3) and the increased release of lactate dehydrogenase (LDH), interleukin (IL)-1β and IL-18. The silencing of GSDMD by small interfering RNA (siRNA) efficiently inhibited the release of LDH and the secretion of IL-1β and IL-18. In addition, rosiglitazone (RGZ) prevented hepatocyte pyroptosis induced by nicotine. Furthermore, we showed that PCB2 attenuated nicotine-induced pyroptosis through the activation of peroxisome proliferator-activated receptor-γ (PPARγ) in hepatocytes. Moreover, administration of PCB2 ameliorated liver injury and hepatocyte pyroptosis in nicotine-treated mice. Hence, our findings demonstrated that PCB2 attenuated pyroptosis and liver damage in a PPARγ-dependent manner. Our results suggest a new mechanism by which PCB2 exerts its liver protective effects.

## 1. Introduction

Tobacco smoking is reported to be one of the leading causes of preventable mortality [1]. The toxicity of tobacco combustion is attributed to a wide variety of products including smoke tars, nitrosamines, aldehydes and heavy metals [2]. Nicotine, a natural active alkaloid, is the addictive component of tobacco and e-cigarettes (an alternative to conventional cigarettes) [3]. It has been reported that nicotine plays a critical role in the development of many diseases, such as cardiovascular disorders and lung cancer [4]. Since clinical trials indicate that smoking is associated with the pathogenesis of hepatic diseases, including non-alcoholic fatty liver disease (NAFLD), hepatitis C and liver cancer [5,6,7,8,9], a quantity of studies focus on the hepatoxicity effect of nicotine. Bandyopadhyaya et al. reported that nicotine induced DNA damage and decreased the contents of total DNA in the liver tissues of Wistar rats, suggesting a nicotine-promoted genotoxicity [10]. In addition, EI-Sokkary et al. revealed the increased level of lipid peroxidation and the reduced activity of superoxide dismutase in the liver of chronic nicotine-administered Sprague Dawley rats [11]. Similarly, a recent study has reported that nicotine increases hepatic cytochrome p-450 level and oxidative stress, as well as the dilation and congestion of central veins in the liver of Sprague Dawley rats [12].

Pyroptosis is a type of the inflammation-associated programmed cell death, which is initially observed in *Salmonella*-infected macrophages [13]. Following studies revealed that the gasdermin family proteins function as pore-forming effectors, leading to cell membrane permeabilization and pyroptosis [14,15]. Recent studies have demonstrated the crucial role of pyroptosis in liver diseases. For instance, by using the hyperactive NLRP3 knock-in mice, Wree et al. revealed that the activation of hepatocyte NLRP3 inflammasome resulted in pyroptotic cell death and consequent liver inflammation and fibrosis [16]. Khanova et al. found that the activation of Caspase-11/4 induced the cleavage of GSDMD, thus triggering pyroptosis in alcoholic hepatitis liver in mice and patients [17]. It has been reported that GSDMD-mediated lipogenesis and NF-ĸB signaling play a key role in the development of non-alcoholic steatohepatitis; GSDMD-NT, the activation form of GSDMD, is positively correlated with liver fibrosis and NAFLD [18]. In addition, the activation of NLRP3 inflammasome leads to pyroptotic cell death of hepatocytes to release NLRP3 inflammasome particles, which are internalized by adjacent hepatic stellate cells, activating them to induce liver fibrosis [19]. Furthermore, the activation of Caspase-4 in Apaf-1 pyroptosome triggers the cleavage of GSDME, therefore inducing pyroptosis and subsequent cholestatic liver failure [20]. Recent reports have indicated that nicotine promotes pyroptosis in macrophages and endothelial cells [21,22,23]. However, whether nicotine could induce hepatocytes to undergo pyroptotic cell death and, if so, what the underlying molecular mechanisms are remain uncharacterized.

Procyanidins are a type of flavonoid polyphenols, which are widely distributed in plant food such as fruits, vegetables, beans and teas [24]. PCB2, composed by 2 flavan-3-ol (−)-epicatechin, is the most ubiquitous procyanidins and has been shown to possess pharmacological properties [25]. Accumulating evidence demonstrates the hepatoprotective effects of PCB2. For instance, Feng et al. have shown that PCB2 suppresses the development of liver fibrosis through the inhibition of the hedgehog signaling pathway [26]. PCB2 increases TFEB-mediated lysosomal activation and decreases oxidative stress, thereby ameliorating free fatty acids-induced hepatic steatosis [25]. It has been reported that PCB2 protects against carbon tetrachloride-caused hepatic damage via the enhancement of the antioxidative system and the downregulation of the inflammatory response [27]. In addition, our previous study has demonstrated that PCB2 suppresses the activation of NLRP3 inflammasome in endothelial cells, suggesting that PCB2 may have a potential in inhibiting pyroptosis [28]. However, whether PCB2 affects hepatocyte pyroptosis remains to be elucidated. To this end, we examined the role of nicotine in hepatocyte pyroptosis, and we also identified the potential effects of PCB2 on nicotine-triggered pyroptotic cell death and the underlying molecular mechanisms.

## 2. Materials and Methods

### 2.1. Reagents, Antibodies and Plasmids

Nicotine was from Cayman (Ann Arbor, MI, USA). PCB2, RGZ, GW9662, Hoechst 33342 and PI were from Sigma (St. Louis, MO, USA). Antibodies against Gasdermin D (39754), HMGB1 (6893), Cleaved-Caspase-1 (4199) and NLRP3 (15101) were from Cell Signaling Technology (Danvers, MA, USA). Antibody against Vinculin (V-4505) was obtained from Sigma. Flag-Gsdmd-FL (80950), Flag-Gsdmd-NT (80951) and Flag-Gsdmd-NT 4A (80952) were from Addgene (Watertown, MA, USA).

### 2.2. Animal Procedure

Male C57BL/6J mice aged 8 weeks were obtained from Vital River Laboratories (Beijing, China). Mice received one week of acclimatization and allocated into four groups at random: the vehicle group, the nicotine group, the PCB2 group and the nicotine plus PCB2 group (*n* = 6 per group). PCB2 (50 mg/kg/day) or saline was daily administrated through oral gavage for 6 weeks. After 2 weeks of PCB2 administration, mice received once-daily intraperitoneal (IP) injections of nicotine (2 mg/kg/day) or saline for 4 weeks. The processes of animal care and experiments were authorized by the Animal Research Committee of Xi’an Jiaotong University (approval no. 2021-1723), and all animal care complied with the National Institutes of Health *Guide for the Care and Use of Laboratory Animals*. For sacrifice, mice were euthanized using the CO_2_ method in accordance with the 2013 AVMA guidelines.

### 2.3. Cell Culture, Transfection and RNA Interference

Human hepatocyte LO2 cells were obtained from ATCC (Manassas, VA, USA) and cultured in DMEM and incubated with 100 μM nicotine for 24 h to induce pyroptosis. Plasmids and siRNA were transfected into LO2 cells by using Lipofectamine 3000 (Invitrogen, Carlsbad, CA, USA) following the manufacturer’s instructions. The siRNA sequences targeting human GSDMD were as follows: siGSDMD-A, 5′-GUGUGUCAACCUGUCUAUCAATT-3′ (forward) and 5′-UUGAUAGACAGGUUGACACACTT-3′ (reverse); siGSDMD-B, 5′-CAGCACCUCAAUGAAUGUGUATT-3′ (forward) and 5′-UACACAUUCAUUGAGGUGCUGTT-3′ (reverse).

### 2.4. Immunoblotting

Cells and mouse liver tissues were homogenized and centrifuged at 12,000× *g* for 10 min to extract proteins. A BCA protein assay kit (ThermoFisher, Waltham, MA, USA) was used to calculate the protein concentrations. The proteins were separated by SDS-PAGE gels, transferred to PVDF membranes and immunoblotted with indicated antibodies.

### 2.5. Caspase-1 Activity Assay

The activity of Caspase-1 was determined by using Caspase-1 activity assay kit (Beyotime, Shanghai, China). Briefly, LO2 cells or 20 mg mouse liver tissues were homogenized at 4 °C. After centrifuge, Ac-YVAD-pNA (acetyl-Tyr-Val-Ala-Asp p-nitroanilide), a substrate of Caspase-1, were incubated with the supernatants at 37 °C for 2 h. The production of yellow pNA was measured at 405 nm with a spectrophotometer.

### 2.6. LDH Release Assay

The activity of LDH was determined by using an LDH cytotoxicity assay kit (ThermoFisher Scientific) following the manufacturer’s instructions. The absorbance at 490 nm was measured using a spectrophotometer. Cytotoxicity was calculated against the absorbance of LDH releasing reagent treated cells.

### 2.7. Cell Death Assay

Necrotic cell death was examined with Hoechst 33342/PI staining. LO2 cells were plated in 6-well plate (1 × 10^5^ cells/well). The cells were incubated with a mixed solution of Hoechst 33342 (0.5 μg/mL) and PI (1 μg/mL) in a 37 °C incubator for 20 min. The fluorescence imaging was taken with a confocal microscope (E-C2; Nikon, Japan). ImageJ software was used for the quantification of PI-positive cells.

### 2.8. Enzyme-Linked Immunosorbent Assay (ELISA)

The concentrations of IL-1β and IL-18 in mouse serums and cultured medium of hepatocytes were determined by ELISA kits (Elabscience, Wuhan, China) following the manufacturer’s protocols.

### 2.9. Cell Viability Assay

Cell viability was measured with CCK-8 assay and MTT assay. For CCK-8 assay, LO2 cells were cultured in 96-well plate, cell viability was determined using cell counting kit-8 (Sigma) in accordance with the manufacturer’s instructions. Absorbance was measured at 450 nm using a spectrophotometer. For MTT assay, LO2 cells were seeded in 96-well plate, cell viability was determined using MTT cell proliferation and cytotoxicity assay kit (Beyotime) following the manufacturer’s instructions.

### 2.10. Statistical Analysis

The statistical differences were analyzed with Student’s *t*-test (evaluations of two groups) or one-way ANOVA (comparisons among multiple groups) followed by Bonferrioni post-test and expressed as mean ± SD. *p* < 0.05 was considered statistically significant.

## 3. Results

### 3.1. Nicotine Triggers Hepatocyte Pyroptosis through the Cleavage of GSDMD

**Figure 1:**
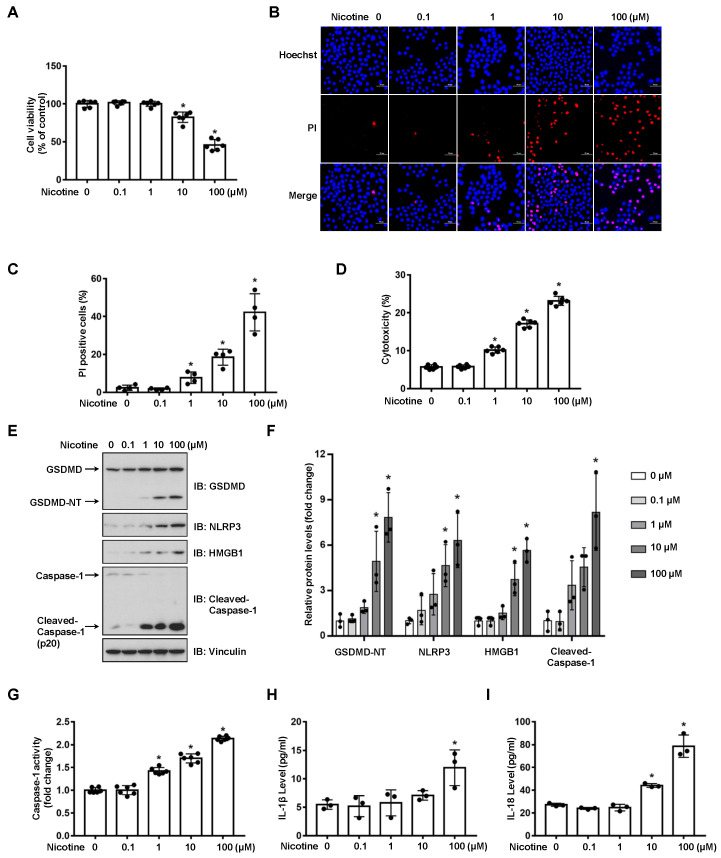
Nicotine induces pyroptosis in hepatocytes. LO2 cells were treated with nicotine at the indicated concentrations for 24 h. (**A**) Cell viability was detected by CCK-8 assay following exposure to nicotine (*n* = 6). (**B**,**C**) Hoechst 33342/PI staining, fluorescence imaging (**B**) was acquired by confocal microscopy. Scale bar: 50 μm. The percentage of PI-positive cells (**C**) was analyzed by using ImageJ software (*n* = 4). (**D**) Cytotoxicity was determined by LDH release assay following exposure to nicotine (*n* = 6). (**E**) The proteins levels of GSDMD, NLRP3, HMGB1 and Cleaved-Caspase-1 was measured by using Western blotting. (**F**) Quantification of GSDMD-NT, NLRP3, HMGB1 and Cleaved-Caspase-1 levels as in (**E**) (*n* = 3). (**G**) The activity of Caspase-1 was determined by using Caspase-1 activity assay kit (*n* = 6). (**H**,**I**) The concentrations of IL-1β (**H**) and IL-18 (**I**) in the conditioned medium were determined by ELISA (*n* = 6). Data shown represent the mean ± SD (*, *p* < 0.05).

To examine the cytotoxicity of nicotine on hepatocytes, LO2 cells were exposed to nicotine at various concentrations. As shown in Figure 1A and Figure S1, nicotine triggered cell death in a dose-dependent manner. Next, we performed Hoechst 33342/PI double staining to discriminate between apoptotic cell death and necrotic cell death after hepatocytes exposed to nicotine. Our results showed that nicotine triggered necrotic cell death as indicated by the enhancement of PI positive staining rate (Figure 1B,C). In addition, LDH release was also enhanced in a concentration-dependent manner (Figure 1D). Both of pyroptosis and necroptosis are lytic types of necrotic cell death, but they require different membrane pore formation proteins [29]. To ascertain whether nicotine induces pyroptotic cell death, we treated LO2 cells with different doses of nicotine. The protein abundance of GSDMD-NT was increased upon the nicotine treatment (Figure 1E,F). Pyroptosis is mediated by inflammasome and Caspase activation, immunoblotting exhibited that nicotine up-regulated the protein abundances of NLRP3 and cleaved-Caspase-1, as well as high mobility group box protein 1 (HMGB1) (Figure 1E,F). Consistently, the activity of Caspase-1 was increased upon the nicotine exposure (Figure 1G). To further illustrate nicotine-promoted hepatocyte pyroptosis, we tested the levels of IL-1β and IL-18 in the conditioned medium by ELISA. The concentrations of these two inflammatory cytokines were also augmented following nicotine stimulation (Figure 1H,I). These results indicated that nicotine promoted hepatocyte pyroptosis in a concentration-dependent manner.

**Figure 2:**
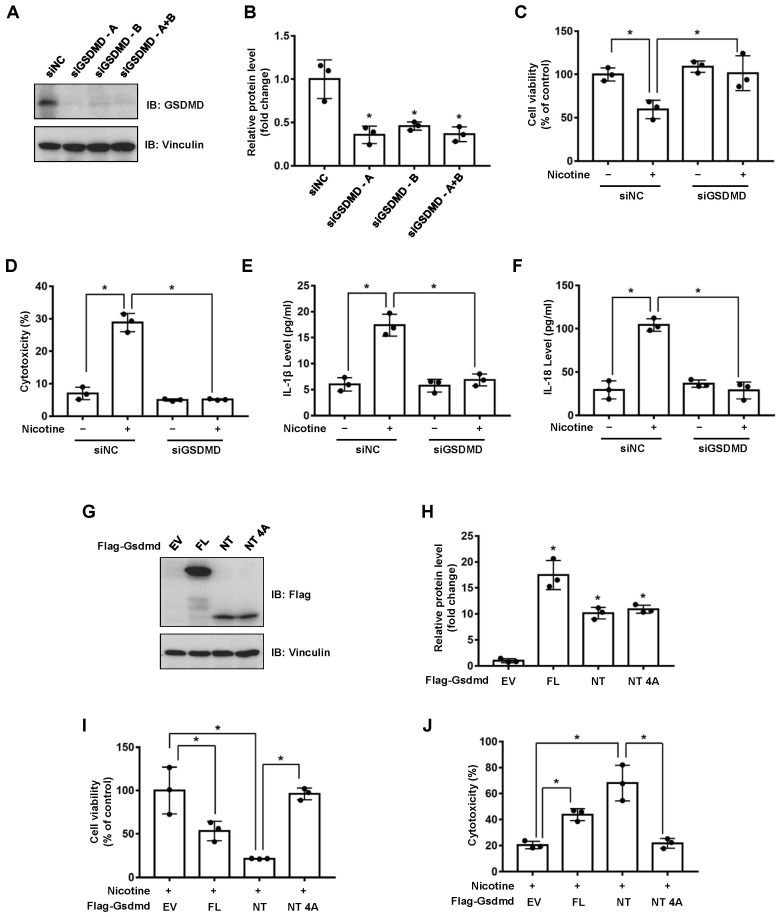
Nicotine induces pyroptosis through the cleavage of GSDMD. (**A**) LO2 cells were transfected with the indicated siRNA for 36 h, the protein levels of GSDMD were determined by using Western blotting. (**B**) Quantification of GSDMD protein level as in (**A**). (**C**–**F**) LO2 cells were transfected with the indicated siRNA for 24 h followed by the treatment with 100 μM nicotine for 24 h. Cell viability was determined by CCK-8 assay following exposure to nicotine (**C**). Cytotoxicity was determined by LDH release assay following exposure to nicotine (**D**). The concentrations of IL-1β (**E**) and IL-18 (**F**) in the conditioned medium were determined by ELISA. (**G**) Western blotting of cell lysates derived from LO2 cells transfected with the indicated plasmids for 36 h. (**H**) Quantification of protein levels as in (**G**). (**I**,**J**) LO2 cells transfected with the indicated plasmids for 24 h followed by the incubation with 100 μM nicotine for 24 h. Cell viability was detected by CCK-8 assay following exposure to nicotine (**I**). Cytotoxicity was determined by LDH release assay following exposure to nicotine (**J**). *n* = 3, data shown represent the mean ± SD (*, *p* < 0.05).

The gasdermins are a protein family causing membrane pore formation and lytic pro-inflammatory cell death [14]. To test whether the nicotine-induced pyroptosis is a GSDMD-dependent type of cell death, we silenced the expression of endogenous GSDMD in LO2 cells (Figure 2A,B). The depletion of GSDMD significantly attenuated cell viability impaired by nicotine (Figure 2C). Moreover, silencing of GSDMD inhibited the release of LDH and the generations of IL-1β and IL-18 (Figure 2D–F). It has been revealed that the cleavage of GSDMD plays a crucial role in pyroptotic cell death [30]. We showed that ectopic expression of mouse Gsdmd-NT (N-terminal cleavage product of Gsdmd-FL), but not Gsdmd-NT 4A (Gsdmd-NT mutant, loss of oligomerization and pore formation function [30]), significantly increased cell death in the context of nicotine treatment (Figure 2G–J). These data therefore revealed that nicotine-induced pyroptosis was dependent on the cleavage of GSDMD.

### 3.2. Activation of PPARγ Prevents Nicotine-Triggered Pyroptosis

**Figure 3:**
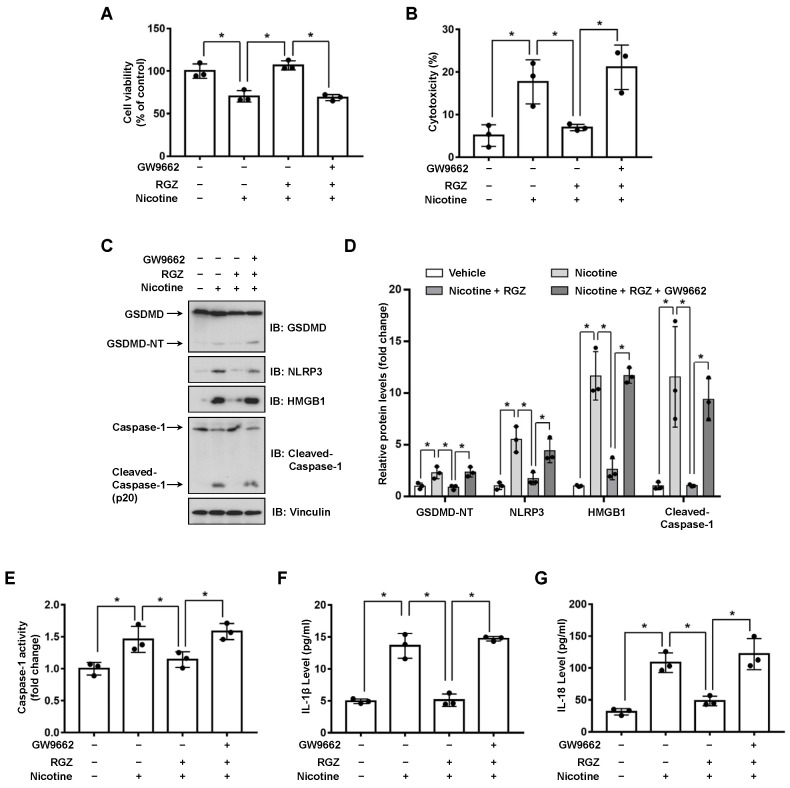
PPARγ activation attenuates nicotine-induced pyroptosis in hepatocytes. LO2 cells were treated with or without 10 μM GW9662 for 1 h followed by the treatment with 10 μM RGZ for 24 h. Cells were exposed to 100 μM nicotine for 24 h before harvesting. (**A**) Cell viability was detected by CCK-8 assay following exposure to nicotine. (**B**) Cytotoxicity was determined by LDH release assay following exposure to nicotine. (**C**) The proteins levels of GSDMD, NLRP3, HMGB1 and Cleaved-Caspase-1 by using Western blotting. (**D**) Quantification of GSDMD-NT, NLRP3, HMGB1 and Cleaved-Caspase-1 levels as in (**C**). (**E**) The activity of Caspase-1 was determined by using Caspase-1 activity assay kit. (**F**,**G**) The concentrations of IL-1β (**F**) and IL-18 (**G**) in the conditioned medium were determined by ELISA. *n* = 3, data shown represent the mean ± SD (*, *p* < 0.05).

PPARs regulate the expression of genes involved in glycolipid metabolism, differentiation and inflammation [31]. To investigate whether PPARs might attenuate nicotine-induced pyroptosis, LO2 cells were incubated with the agonist of PPARγ or PPARδ before nicotine treatment. As shown in Figure S2A,B and Figure 3C,D, RGZ (a selective agonist of PPARγ) but not GW501516 (a synthetic ligand of PPARδ), decreased the protein abundance of NLRP3 and the cleavage of GSDMD caused by nicotine. To confirm the hypothesis that RGZ suppresses pyroptosis through the activation of PPARγ. LO2 cells were exposed to GW9662, an antagonist of PPARγ, followed by the treatment of RGZ and nicotine. As shown in Figure 3A, GW9662 blocked the beneficial effect of RGZ on nicotine-impaired cell viability. In addition, in the presence of GW9662, RGZ failed to inhibit the elevated release of LDH (Figure 3B) and the activation of Caspase-1 induced by nicotine (Figure 3E). Moreover, nicotine-triggered secretion of inflammatory cytokines was reduced by RGZ whereas GW9662 successfully eliminated this effect (Figure 3F,G). These results indicated that PPARγ activation attenuated nicotine-induced hepatocyte pyroptosis.

### 3.3. PCB2 Attenuates Nicotine-Induced Pyroptosis in Hepatocytes

**Figure 4:**
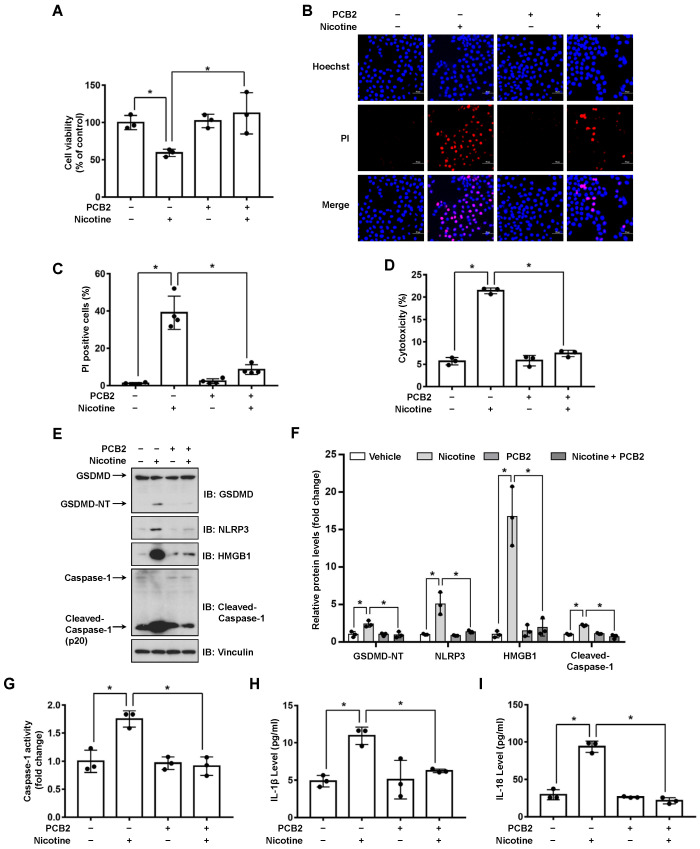
PCB2 prevents nicotine-induced pyroptosis in hepatocytes. LO2 cells were treated with 10 μM PCB2 for 24 h followed by the incubation with 100 μM nicotine for 24 h before harvesting. (**A**) Cell viability was detected by CCK-8 assay following exposure to nicotine (*n* = 3). (**B**,**C**) Hoechst 33342/PI staining, fluorescence imaging (**B**) was acquired by confocal microscopy. Scale bar: 50 μm. The percentage of PI-positive cells (**C**) was analyzed by using ImageJ software (*n* = 4). (**D**) Cytotoxicity was determined by LDH release assay following exposure to nicotine (*n* = 3). (**E**) The proteins levels of GSDMD, NLRP3, HMGB1 and Cleaved-Caspase-1 by using immunoblotting. (**F**) Quantification of GSDMD-NT, NLRP3, HMGB1 and Cleaved-Caspase-1 levels as in (**E**) (*n* = 3). (**G**) The activity of Caspase-1 was determined by using Caspase-1 activity assay kit (*n* = 3). (**H**,**I**) The concentrations of IL-1β (**H**) and IL-18 (**I**) in the conditioned medium were determined by ELISA (*n* = 3). Data shown represent the mean ± SD (*, *p* < 0.05).

It has been revealed that the extracts or pure compounds derived from natural products exhibit anti-inflammatory properties [32]. Next, we decided to test whether natural products are advantageous in preventing nicotine-caused pyroptosis. By examining several natural products with anti-inflammatory activities, we observed that PCB2 mitigated nicotine-promoted the cleavage of GSDMD in LO2 hepatocytes (Figure S3). To further elucidate the possible role of PCB2 on nicotine-triggered hepatocyte pyroptosis, LO2 cells were incubated with PCB2 before the exposure to nicotine. As shown in Figure 4A, PCB2 ameliorated the impaired cell viability caused by nicotine treatment. In addition, the PI-positive cells and LDH release were increased after nicotine exposure, PCB2 attenuated the cell death provoked by nicotine (Figure 4B–D). Furthermore, immunoblotting showed that PCB2 decreased the cleavage of GSDMD and Caspase-1 triggered by nicotine (Figure 4E,F). Moreover, PCB2 also diminished the activity of Caspase-1 (Figure 4G) and the concentration of pyroptosis response cytokines, including IL-1β and IL-18 (Figure 4H,I). Thus, we demonstrated that PCB2 effectively prevented LO2 cells from pyroptotic cell death triggered by nicotine.

### 3.4. PPARγ Antagonist Abrogates the Effects of PCB2 on Pyroptosis

**Figure 5:**
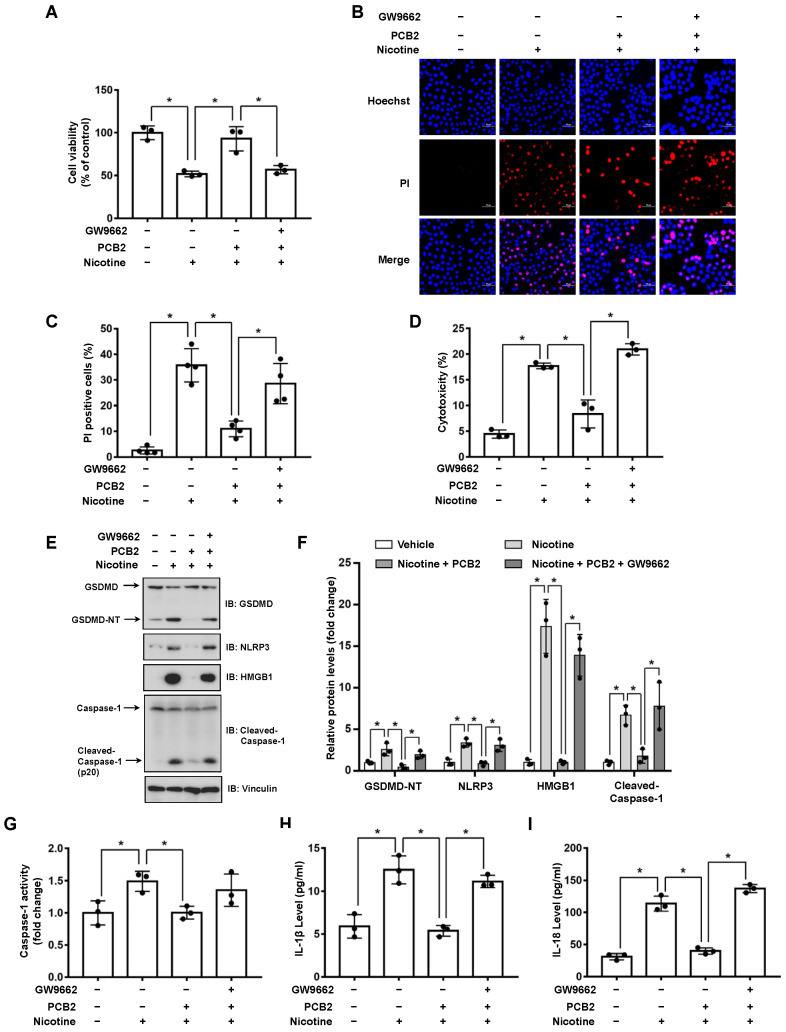
PCB2 prevents nicotine-induced pyroptosis through the activation of PPARγ. LO2 cells were treated with or without 10 μM GW9662 for 1 h followed by the treatment with 10 μM PCB2 for 24 h. Cells were exposed to 100 μM nicotine for 24 h before harvesting. (**A**) Cell viability was detected by CCK-8 assay following exposure to nicotine (*n* = 3). (**B**,**C**) Hoechst 33342/PI staining, fluorescence imaging (**B**) was acquired by confocal microscopy. Scale bar: 50 μm. The percentage of PI-positive cells (**C**) was analyzed by using ImageJ software (*n* = 4). (**D**) Cytotoxicity was determined by LDH release assay following exposure to nicotine (*n* = 3). (**E**) The proteins levels of GSDMD, NLRP3, HMGB1 and Cleaved-Caspase-1 by using immunoblotting. (**F**) Quantification of GSDMD-NT, NLRP3, HMGB1 and Cleaved-Caspase-1 levels as in (**E**) (*n* = 3). (**G**) The activity of Caspase-1 was determined by using Caspase-1 activity assay kit (*n* = 3). (**H**,**I**) The concentrations of IL-1β (**H**) and IL-18 (**I**) in the conditioned medium were determined by ELISA (*n* = 3). Data shown represent the mean ± SD (*, *p* < 0.05).

We recently showed that PCB2 induced macrophage M2 polarization through a PPARγ-dependent mechanism [33]. Thus, we hypothesized that PCB2 might mitigate pyroptosis via PPARγ activation. To this end, LO2 cells were pretreated with GW9662 and then with PCB2 before the exposure to nicotine in the presence of PCB2. We found that PCB2 failed to rescue nicotine-impaired cell viability in the presence of GW9662 (Figure 5A). Consistently, in the context of GW9662, PCB2 failed to decrease the number of PI-positive cells, the cleavage of GSDMD and Caspase-1, the activity of LDH and the release of inflammatory cytokines (Figure 5B–I). These results demonstrated that PCB2 attenuated nicotine-induced hepatocyte pyroptosis via a PPARγ-dependent mechanism.

### 3.5. PCB2 Attenuates Liver Injury and Hepatocyte Pyroptosis in Mice

**Figure 6:**
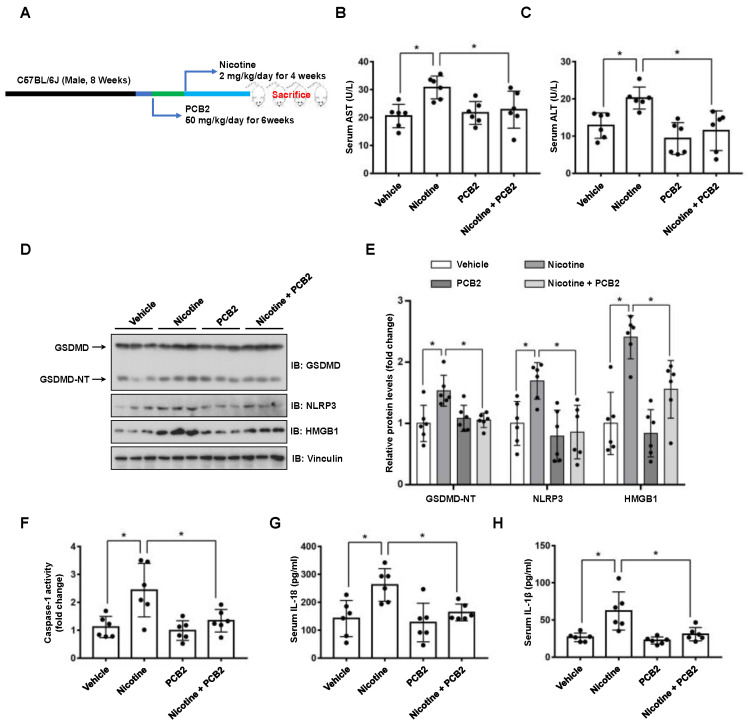
PCB2 ameliorates nicotine-induced pyroptosis and liver damage in mice. C57BL/6J mice were administrated with PCB2 (50 mg/kg/day) or saline for 6 weeks. After 2 weeks of PCB2 administration, mice were received daily IP injection of nicotine (2 mg/kg/day) or saline for 4 weeks. (**A**) A schematic illustration of the animal treatment procedure. (**B**) Serum levels of AST. (**C**) Serum levels of ALT. (**D**) Immunoblot analysis of the protein levels of GSDMD, NLRP3 and HMGB1 in liver tissues of the mice. (**E**) Quantification of GSDMD-NT, NLRP3 and HMGB1 levels as in (**D**). (**F**) The activity of Caspase-1 in liver tissues of the mice. (**G**,**H**) The concentrations of IL-1β (**G**) and IL-18 (**H**) in the serum of the mice. *n* = 6 per group; Data shown represent the mean ± SD (*, *p* < 0.05).

To validate these in vitro findings, we further examined the beneficial effects of PCB2 in mice (Figure 6A). Compared with the vehicle group, mice in the nicotine group had gained less weight; PCB2 ameliorated the weight loss (Figure S4). Liver injury markers, serum AST and ALT, were also analyzed. As shown in Figure 6B,C, compared with the vehicle group, the nicotine group had higher serum levels of AST and ALT, which were significantly increased by PCB2 administration. These results suggested that PCB2 attenuated weight loss and liver injury in nicotine-treated mice. Furthermore, immunoblotting showed that the protein abundance of GSDMD-NT, NLRP3 and HMGB1 were increased in the livers of nicotine group, whereas PCB2 administration to nicotine-treated mice decreased the expression of GSDMD-NT, NLRP3 and HMGB1 (Figure 6D,E). Consistently, nicotine increased the activity of Caspase-1 and the serum levels of inflammatory cytokines, PCB2 treatment mitigated these effects compared with nicotine group (Figure 6F–H). These data together supported the notion that PCB2 inhibited pyroptosis in the liver of nicotine-treated mice.

## 4. Discussion

The current study provided experimental evidence that PCB2 inhibited hepatocyte pyroptosis, thus ameliorating nicotine-induced liver injury. Our findings are as follows: (1) nicotine promoted the pyroptotic cell death of hepatocytes through the cleavage of GSDMD; (2) PCB2 attenuated hepatocyte pyroptosis and liver damage caused by nicotine; (3) the suppressive effect of PCB2 on pyroptosis was mediated via the activation of PPARγ.

Inflammasome complexes are assembled in response to various stimuli. Caspases are activated by inflammasomes or directly activated by the stimuli. The active Caspases process gasdermins to constitute cell membrane pores, triggering pyroptotic cell death and the release of cytosolic content such as LDH, HMGB1, IL-1β, IL-18 and even the entire inflammasome complexes. Several recent studies have indicated that nicotine-induced pyroptotic cell death contributes to the progression of atherosclerosis. Nicotine promotes atherosclerosis through the induction of NLRP3-dependent macrophage pyroptosis; HDAC6 depletion abolishes nicotine-caused pyroptosis by inhibiting NLRP3 transcription [22]. Another study in macrophages indicates that the increased level of ROS by nicotine induces the activation of TXNIP-NLRP3 inflammasome signaling, thereby promoting pyroptosis and exacerbating atherosclerosis [21]. In addition, the pro-pyroptosis effect of nicotine is attributed to its ability to up-regulate ROS generation and Caspase-1 activity in endothelial cells [23]. In this study, we revealed that nicotine induced pyroptosis in hepatocytes, featured by the increased level of PI positive rate, LDH release, IL-1β and IL-18, as well as the activation of GSDMD and Caspase-1 (Figure 1). These findings therefore demonstrated that nicotine triggers pyroptosis in hepatocytes.

The gasdermin family proteins are identified pyroptosis executors that cause cell membrane pore-forming and permeabilization. Structurally, gasdermins contain two distinct domains, which can be cleaved by Caspases. GSDMD and GSDME-induced pyroptosis have been well documented. The activation of GSDMD is regulated by Caspase-1 or Caspase-4/5/11 [34,35], apoptotic Caspase-8 also has been reported to induced GSDMD cleavage [36]. On the other hand, the cleavage of GSDME by Caspase-3 has been demonstrated to induce pyroptotic cell death [37]. It is noteworthy that the N-terminal of other gasdermin family members, such as GSDMA, GSDMB and GSDMC, also have a pore-formation function and can induce pyroptosis [38], although their upstream signaling remains largely uncharacterized. Our data showed that depletion of endogenous GSDMD inhibited nicotine-promoted pyroptosis (Figure 2A–F), indicating that nicotine induced hepatocyte pyroptosis via a GSDMD-dependent mechanism. In addition, ectopic expression of Gsdmd-NT increased the activity of LDH and decreased the viability of hepatocytes (Figure 2G–J), indicating that the cleavage of GSDMD plays a critical role in nicotine-caused pyroptosis. However, whether other gasdermins participate in nicotine-induced hepatocyte pyroptosis warrants further investigation.

Both acute and chronic liver injury may induce wound-healing response, leading to the formation of hepatic fibrosis even hepatic carcinoma [39]. It has been reported that chronic inhalation of electronic cigarettes vapor containing nicotine increases the level of circulating inflammatory cytokines and induces liver fibrosis in mice [40]. Compared with saline aerosol control, ApoE(−/−) mice exposed to electronic nicotine delivery systems exhibited hepatic lipid accumulation and oxidative stress, as well as increased hepatocyte apoptosis [3]. In addition, animal studies suggest that chronic nicotine administration leads to liver injury as indicated by the increased level of oxidative stress, DNA damage, transaminases and triglyceride accumulation in the liver [10,11,12,41,42]. Consistently, our data demonstrated that 4 weeks of nicotine exposure led to mouse liver damage (Figure 6B,C). Given the crucial role of pyroptosis in liver injury [16,17,18,19] and our in vitro and in vivo results that nicotine promoted hepatocyte pyroptosis, it is likely that nicotine-induced hepatocyte pyroptosis may contribute to the progression of liver injury.

A previous report has indicated that PPARγ plays a crucial role in protecting liver from diverse diseases such as hepatitis, liver fibrosis, liver cancer and NAFLD [43]. Although PPARγ agonist troglitazone showed severe hepatotoxicity, studies pointed out that the liver toxicity was associated with its exclusive side chain [44]. RGZ does not possess the hepatotoxic property of troglitazone and has no effect on inducing apoptosis of hepatocytes [45,46]. Conversely, RGZ is reported to prevent Schistosoma japonicum-induced liver fibrosis and reduce the serum level of inflammatory cytokines in mice [47]. Furthermore, RGZ attenuates the activation of NLRP3 inflammasome and subsequent Caspase-1 and IL-1β maturation in macrophage [48], suggesting RGZ may has a potential for the attenuation of pyroptosis. In this study, we demonstrated that RGZ reduced nicotine-caused hepatocyte pyroptotic cell death (Figure 3). More importantly, GW9662 prevented the effect of RGZ on nicotine-induced pyroptosis (Figure 3), indicating that the activation of PPARγ plays a significant role in ameliorating nicotine-induced hepatocyte pyroptosis.

Epidemiological studies indicate that fruit and vegetable consumptions are closely correlated with the reduced risk of hepatic diseases [49,50]. PCB2, a type of flavonoid polyphenols which is enriched in fruits and vegetables, has been shown to protect against liver diseases such as liver fibrosis and NAFLD [26,51]. The beneficial effects of PCB2 could be attributed to its anti-oxidation and ROS scavenging properties. However, other mechanisms might be involved in this process. We previously reported that PCB2 suppressed the detrimental effect of LPS through the inhibition of AP-1 in endothelial cells [28]. Consistently, our current study showed that PCB2 decreased nicotine-induced NLRP3 protein level and its downstream Caspase-1 maturation (Figure 4E,F). In addition, it has been reported that PCB2 promotes nuclear location of Nrf2 to induce the expression of PPARγ [52]. Another study points out that PPARγ suppresses NLRP3 inflammasome activation through binding with NLRP3 and disrupting the assemble of NLRP3 inflammasome complex [48]. We found that GW9662 inhibited the effect of PCB2 on nicotine-triggered pyroptosis (Figure 5), suggesting the inhibitory effect of PCB2 on NLRP3 inflammasome activation and subsequent pyroptosis is dependent on PPARγ activation. However, whether PCB2 promotes the interaction between PPARγ and NLRP3 to suppress pyroptosis warrants further investigation.

## 5. Conclusions

In conclusion, our results reveal a novel role of nicotine in hepatocyte pyroptotic cell death. Our findings also provide new insights into the mechanism by which PCB2 exerts its anti-pyroptotic effects and hepatic benefits through the activation of PPARγ, highlighting the beneficial effects of dietary procyanidins against hepatic diseases.

## Data Availability

All relevant data are within the manuscript and its Supporting Information files.

## Supplementary Materials

The following supporting information can be downloaded at: https://www.mdpi.com/article/10.3390/nu14091756/s1, Figure S1: Nicotine decreased cell viability of hepatocytes; Figure S2: Rosiglitazone (RGZ) prevents nicotine-induced pyroptosis in hepatocytes; Figure S3: Procyanidin B2 prevents nicotine-induced pyroptosis in hepatocytes; Figure S4: PCB2 ameliorated nicotine-induced weight loss.

## References

[1] Frieden T.R. (2017). A Safer, Healthier U.S.: The Centers for Disease Control and Prevention, 2009–2016. Am. J. Prev. Med..

[2] Long G.A. (2014). Comparison of select analytes in exhaled aerosol from e-cigarettes with exhaled smoke from a conventional cigarette and exhaled breaths. Int. J. Environ. Res. Public Health.

[3] Hasan K.M., Friedman T.C., Shao X., Parveen M., Sims C., Lee D.L., Espinoza-Derout J., Sinha-Hikim I., Sinha-Hikim A.P. (2019). E-cigarettes and Western Diet: Important Metabolic Risk Factors for Hepatic Diseases. Hepatology.

[4] Thorgeirsson T.E., Geller F., Sulem P., Rafnar T., Wiste A., Magnusson K.P., Manolescu A., Thorleifsson G., Stefansson H., Ingason A. (2008). A variant associated with nicotine dependence, lung cancer and peripheral arterial disease. Nature.

[5] Zimmermann T., Hueppe D., Mauss S., Buggisch P., Pfeiffer-Vornkahl H., Grimm D., Galle P.R., Alshuth U. (2016). Effects of Smoking on Pegylated Interferon alpha 2a and First Generation Protease Inhibitor-based Antiviral Therapy in Naive Patients Infected with Hepatitis C Virus Genotype 1. J. Gastrointest. Liver Dis. JGLD.

[6] Zein C.O., Unalp A., Colvin R., Liu Y.C., McCullough A.J., Nonalcoholic Steatohepatitis Clinical Research N. (2011). Smoking and severity of hepatic fibrosis in nonalcoholic fatty liver disease. J. Hepatol..

[7] Zhao J.K., Wu M., Kim C.H., Jin Z.Y., Zhou J.Y., Han R.Q., Yang J., Zhang X.F., Wang X.S., Liu A.M. (2017). Jiangsu Four Cancers Study: A large case-control study of lung, liver, stomach, and esophageal cancers in Jiangsu Province, China. Eur. J. Cancer Prev. Off. J. Eur. Cancer Prev. Organ..

[8] Karagozian R., Baker E., Houranieh A., Leavitt D., Baffy G. (2013). Risk profile of hepatocellular carcinoma reveals dichotomy among US veterans. J. Gastrointest. Cancer.

[9] El-Zayadi A., Selim O., Hamdy H., El-Tawil A., Badran H.M., Attia M., Saeed A. (2004). Impact of cigarette smoking on response to interferon therapy in chronic hepatitis C Egyptian patients. World J. Gastroenterol..

[10] Bandyopadhyaya G., Sinha S., Chattopadhyay B.D., Chakraborty A. (2008). Protective role of curcumin against nicotine-induced genotoxicity on rat liver under restricted dietary protein. Eur. J. Pharmacol..

[11] El-Sokkary G.H., Cuzzocrea S., Reiter R.J. (2007). Effect of chronic nicotine administration on the rat lung and liver: Beneficial role of melatonin. Toxicology.

[12] Khaled S., Makled M.N., Nader M.A. (2020). Tiron protects against nicotine-induced lung and liver injury through antioxidant and anti-inflammatory actions in rats in vivo. Life Sci..

[13] Brennan M.A., Cookson B.T. (2000). Salmonella induces macrophage death by caspase-1-dependent necrosis. Mol. Microbiol..

[14] Broz P., Pelegrin P., Shao F. (2020). The gasdermins, a protein family executing cell death and inflammation. Nat. Rev. Immunol..

[15] Shi J., Gao W., Shao F. (2017). Pyroptosis: Gasdermin-Mediated Programmed Necrotic Cell Death. Trends Biochem. Sci..

[16] Wree A., Eguchi A., McGeough M.D., Pena C.A., Johnson C.D., Canbay A., Hoffman H.M., Feldstein A.E. (2014). NLRP3 inflammasome activation results in hepatocyte pyroptosis, liver inflammation, and fibrosis in mice. Hepatology.

[17] Khanova E., Wu R., Wang W., Yan R., Chen Y., French S.W., Llorente C., Pan S.Q., Yang Q., Li Y. (2018). Pyroptosis by caspase11/4-gasdermin-D pathway in alcoholic hepatitis in mice and patients. Hepatology.

[18] Xu B., Jiang M.Z., Chu Y., Wang W.J., Chen D., Li X.W., Zhang Z., Zhang D., Fan D.M., Nie Y.Z. (2018). Gasdermin D plays a key role as a pyroptosis executor of non-alcoholic steatohepatitis in humans and mice. J. Hepatol..

[19] Gaul S., Leszczynska A., Alegre F., Kaufmann B., Johnson C.D., Adams L.A., Wree A., Damm G., Seehofer D., Calvente C.J. (2021). Hepatocyte pyroptosis and release of inflammasome particles induce stellate cell activation and liver fibrosis. J. Hepatol..

[20] Xu W., Che Y., Zhang Q., Huang H., Ding C., Wang Y., Wang G., Cao L., Hao H. (2021). Apaf-1 Pyroptosome Senses Mitochondrial Permeability Transition. Cell Metab..

[21] Mao C.Y., Li D.J., Zhou E., Zhang J.F., Wang C.Q., Xue C. (2021). Nicotine exacerbates atherosclerosis through a macrophage-mediated endothelial injury pathway. Aging-US.

[22] Xu S., Chen H.W., Ni H.E., Dai Q.Y. (2021). Targeting HDAC6 attenuates nicotine-induced macrophage pyroptosis via NF-kappa B/NLRP3 pathway. Atherosclerosis.

[23] Wu X., Zhang H., Qi W., Zhang Y., Li J., Li Z., Lin Y., Bai X., Liu X., Chen X. (2018). Nicotine promotes atherosclerosis via ROS-NLRP3-mediated endothelial cell pyroptosis. Cell Death Dis..

[24] Zhu X., Tian X., Yang M., Yu Y., Zhou Y., Gao Y., Zhang L., Li Z., Xiao Y., Moses R.E. (2021). Procyanidin B2 Promotes Intestinal Injury Repair and Attenuates Colitis-Associated Tumorigenesis via Suppression of Oxidative Stress in Mice. Antioxid. Redox Signal..

[25] Su H.M., Li Y.T., Hu D.W., Xie L.H., Ke H.H., Zheng X.D., Chen W. (2018). Procyanidin B2 ameliorates free fatty acids-induced hepatic steatosis through regulating TFEB-mediated lysosomal pathway and redox state. Free Radic. Bio. Med..

[26] Feng J., Wang C., Liu T., Li J., Wu L., Yu Q., Li S., Zhou Y., Zhang J., Chen J. (2019). Procyanidin B2 inhibits the activation of hepatic stellate cells and angiogenesis via the Hedgehog pathway during liver fibrosis. J. Cell. Mol. Med..

[27] Yang B.Y., Zhang X.Y., Guan S.W., Hua Z.C. (2015). Protective Effect of Procyanidin B2 against CCl4-Induced Acute Liver Injury in Mice. Molecules.

[28] Yang H., Xiao L., Yuan Y., Luo X., Jiang M., Ni J., Wang N. (2014). Procyanidin B2 inhibits NLRP3 inflammasome activation in human vascular endothelial cells. Biochem. Pharmacol..

[29] Frank D., Vince J.E. (2019). Pyroptosis versus necroptosis: Similarities, differences, and crosstalk. Cell Death Differ..

[30] Liu X., Zhang Z., Ruan J., Pan Y., Magupalli V.G., Wu H., Lieberman J. (2016). Inflammasome-activated gasdermin D causes pyroptosis by forming membrane pores. Nature.

[31] Gross B., Pawlak M., Lefebvre P., Staels B. (2017). PPARs in obesity-induced T2DM, dyslipidaemia and NAFLD. Nat. Rev. Endocrinol..

[32] Azab A., Nassar A., Azab A.N. (2016). Anti-Inflammatory Activity of Natural Products. Molecules.

[33] Tian Y., Yang C., Yao Q., Qian L., Liu J., Xie X., Ma W., Nie X., Lai B., Xiao L. (2019). Procyanidin B2 Activates PPARgamma to Induce M2 Polarization in Mouse Macrophages. Front. Immunol..

[34] Shi J., Zhao Y., Wang K., Shi X., Wang Y., Huang H., Zhuang Y., Cai T., Wang F., Shao F. (2015). Cleavage of GSDMD by inflammatory caspases determines pyroptotic cell death. Nature.

[35] Kayagaki N., Stowe I.B., Lee B.L., O’Rourke K., Anderson K., Warming S., Cuellar T., Haley B., Roose-Girma M., Phung Q.T. (2015). Caspase-11 cleaves gasdermin D for non-canonical inflammasome signalling. Nature.

[36] Chen K.W., Demarco B., Heilig R., Shkarina K., Boettcher A., Farady C.J., Pelczar P., Broz P. (2019). Extrinsic and intrinsic apoptosis activate pannexin-1 to drive NLRP3 inflammasome assembly. EMBO J..

[37] Wang Y., Gao W., Shi X., Ding J., Liu W., He H., Wang K., Shao F. (2017). Chemotherapy drugs induce pyroptosis through caspase-3 cleavage of a gasdermin. Nature.

[38] Ding J., Wang K., Liu W., She Y., Sun Q., Shi J., Sun H., Wang D.C., Shao F. (2016). Pore-forming activity and structural autoinhibition of the gasdermin family. Nature.

[39] Hernandez-Gea V., Friedman S.L. (2011). Pathogenesis of liver fibrosis. Annu. Rev. Pathol..

[40] Crotty Alexander L.E., Drummond C.A., Hepokoski M., Mathew D., Moshensky A., Willeford A., Das S., Singh P., Yong Z., Lee J.H. (2018). Chronic inhalation of e-cigarette vapor containing nicotine disrupts airway barrier function and induces systemic inflammation and multiorgan fibrosis in mice. Am. J. Physiol. Regul. Integr. Comp. Physiol..

[41] Kuru P., Bilgin S., Mentese S.T., Tazegul G., Ozgur S., Cilingir O.T., Akakin D., Yarat A., Kasimay O. (2015). Ameliorative effect of chronic moderate exercise in smoke exposed or nicotine applied rats from acute stress. Nicotine Tob. Res. Off. J. Soc. Res. Nicotine Tob..

[42] Dangana E.O., Michael O.S., Omolekulo T.E., Areola E.D., Olatunji L.A. (2019). Enhanced hepatic glycogen synthesis and suppressed adenosine deaminase activity by lithium attenuates hepatic triglyceride accumulation in nicotine-exposed rats. Biomed. Pharmacother. Biomed. Pharmacother..

[43] Wu L.W., Guo C.Y., Wu J.Y. (2020). Therapeutic potential of PPAR gamma natural agonists in liver diseases. J. Cell. Mol. Med..

[44] Scheen A.J. (2001). Thiazolidinediones and liver toxicity. Diabetes Metab..

[45] Guo L., Zhang L., Sun Y.M., Muskhelishvili L., Blann E., Dial S., Shi L.M., Schroth G., Dragan Y.P. (2006). Differences in hepatotoxicity and gene expression profiles by anti-diabetic PPAR gamma agonists on rat primary hepatocytes and human HepG2 cells. Mol. Divers..

[46] Bae M.A., Rhee H., Song B.J. (2003). Troglitazone but not rosiglitazone induces G1 cell cycle arrest and apoptosis in human and rat hepatoma cell lines. Toxicol. Lett..

[47] Chen H., He Y.W., Liu W.Q., Zhang J.H. (2008). Rosiglitazone prevents murine hepatic fibrosis induced by Schistosoma japonicum. World J. Gastroenterol..

[48] Yang C.C., Wu C.H., Lin T.C., Cheng Y.N., Chang C.S., Lee K.T., Tsai P.J., Tsai Y.S. (2021). Inhibitory effect of PPARgamma on NLRP3 inflammasome activation. Theranostics.

[49] Wu Y., Li Y., Giovannucci E. (2021). Potential Impact of Time Trend of Lifestyle Risk Factors on Burden of Major Gastrointestinal Cancers in China. Gastroenterology.

[50] Kim S.A., Shin S. (2020). Fruit and vegetable consumption and non-alcoholic fatty liver disease among Korean adults: A prospective cohort study. J. Epidemiol. Commun. H.

[51] Xing Y.W., Lei G.T., Wu Q.H., Jiang Y., Huang M.X. (2019). Procyanidin B2 protects against diet-induced obesity and non-alcoholic fatty liver disease via the modulation of the gut microbiota in rabbits. World J. Gastroenterol..

[52] Liu L., Wang R., Xu R., Chu Y., Gu W. (2022). Procyanidin B2 ameliorates endothelial dysfunction and impaired angiogenesis via the Nrf2/PPARgamma/sFlt-1 axis in preeclampsia. Pharmacol. Res..

