# Supplementary material for: Procyanidin B2 Attenuates Nicotine-Induced Hepatocyte Pyroptosis through a PPARγ-Dependent Mechanism
Source: Nutrients. 2022 Apr 22;14(9):1756. doi: 10.3390/nu14091756 (PMC9103831; doi:10.3390/nu14091756)
Supplement: Supplementary file 1 [file nutrients-14-01756-s001.zip › nutrients-1687737-supplementary.pdf]

Supplementary Materials

Procyanidin B2 attenuates nicotine-induced hepatocytes pyroptosis through a PPARγ-dependent mechanism

Jia Liu, Qinyu Yao, Xinya Xie, Qi Cui, Tingting Jiang, Ziwei Zhao, Xiong Du, Baochang Lai, Lei Xiao, Nanping Wang

Figure S1 (related to Figure 1)

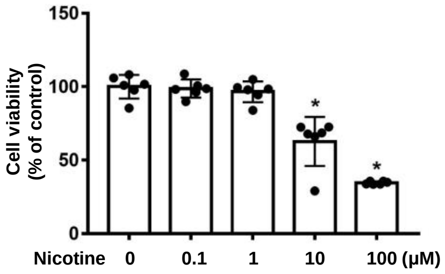

Figure S1. Nicotine decreased cell viability of hepatocytes. LO2 cells were incubated with nicotine at the indicated concentrations for 24 h. Cell viability was determined by MTT assay following exposure to nicotine. Six sets of independent experiments were performed and data shown represent the mean ± SD (*, $p < 0.05$).

Figure S2 (related to Figure 3)

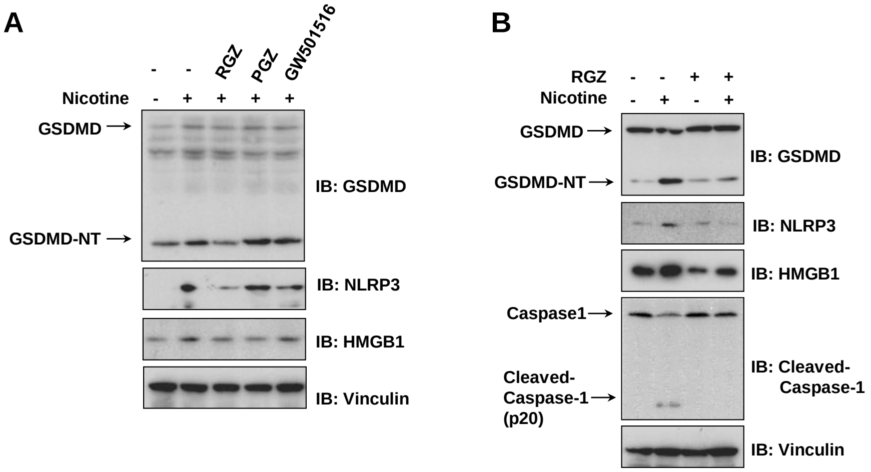

Figure S2. Rosiglitazone (RGZ) prevents nicotine-induced pyroptosis in hepatocytes. (A) Immunoblot analysis of whole cell lysates derived from LO2 cells pretreated with RGZ (10 μM), PGZ (10 μM) or GW501516 (1 μM) for 24 h, and then treated with nicotine (100 μM) for 24 h before harvesting. (B) Immunoblot analysis of whole cell lysates derived from LO2 cells pretreated with RGZ (10 μM) for 24 h, and then treated with nicotine (100 μM) for 24 h before harvesting.

Figure S3 (related to Figure 4)

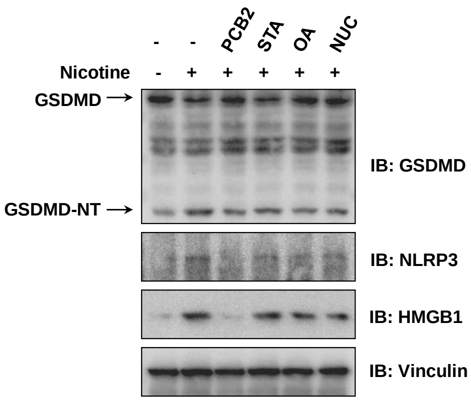

Figure S3. Procyanidin B2 prevents nicotine-induced pyroptosis in hepatocytes. Immunoblot analysis of whole cell lysates derived from LO2 cells pretreated with procyanidin B2 (PCB2, 10 μM), stachydrine (STA, 10 μM), oleanolic acid (OA, 10 μM) or nuciferine (NUC, 10 μM) for 24 h, and then treated with nicotine (100 μM) for 24 h before harvesting.

Figure S4 (related to Figure 6)

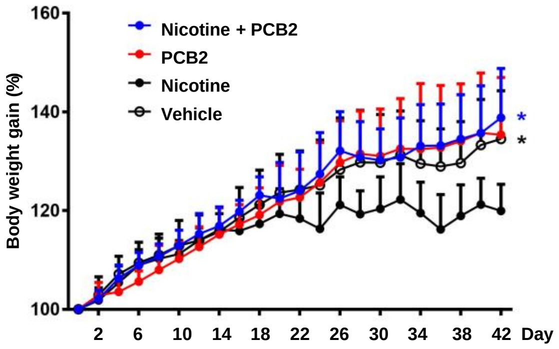

Figure S4. PCB2 ameliorated nicotine-induced weight loss. C57BL/6J mice were administrated with PCB2 (50 mg/kg/day) or saline for 6 weeks. After 2 weeks of PCB2 administration, mice were received daily IP injection of nicotine (2 mg/kg/day) or saline for 4 weeks. The body weight growth rates of the mice. The body weight gain was calculated as follows: $W_n/W_0 \times 100\%$. W_n is body weight in n days, W_0 is the body weight in day 0.
